# Investigation of ion recombination and polarity effects in high dose rate electron beams

**DOI:** 10.1002/acm2.70600

**Published:** 2026-04-28

**Authors:** Ratna Sari Dewi, Mardon Limena, Dwi Seno Kuncoro Sihono, Poonam Yadav, Supriyanto Ardjo Pawiro

**Affiliations:** ^1^ Department of Physics, Faculty of Mathematics and Natural Sciences Universitas Indonesia Depok Indonesia; ^2^ Department of Radiation Oncology Mandaya Royal Puri Hospital Jakarta Indonesia; ^3^ Department of Radiation Oncology Dharmais Cancer Hospital Jakarta Indonesia; ^4^ Feinberg School of Medicine Northwestern University Chicago Illinois USA

**Keywords:** HDRE, ion recombination, ionization chamber, polarity effect

## Abstract

**Background:**

Accurate dosimetry is essential for enhancing the effectiveness and safety of radiotherapy treatment. High dose rate electron (HDRE) beams, a dose rate up to ten times higher at the measurement point than standard electron beam at the same Monitor Unit (MU). The increased ionization density raises concern about ion recombination effects, which may affect the reliability of ionization chamber readings.

**Purpose:**

This study aims to evaluate the effects of ion recombination ($k_{s}$) and polarity ($k_{pol}$) across various dosimetry protocols in order to determine the most suitable approach for HDRE settings.

**Methods:**

Measurements were carried out using Elekta VersaHD, linear accelerator at 6 MeV and 10 MeV both in standard and HDRE modes using three ionization chambers: IBA PPC40, FC65‐P and CC13. Data were collected at a source‐to‐surface distance (SSD) of 100 cm, referring to the standard dosimetry protocols TRS‐398 and AAPM TG‐51, along with a modified method. Modified method using beam quality conversion factor ($k_{Q}^{\prime }$) derived from Monte Carlo simulation and specific adjustment was applied to PPC40, where the Effective Point of Measurement was determined to be 1.39 mm from the window surface.

**Results:**

Based on three dosimetry protocols, all ionization chambers showed improved performance, with the $k_{s}$ values decreasing by approximately 0.4% to 3% as the operational voltage increased from 100 to 400 V, indicating enhanced charge collection and less recombination effects. In contrast, all of $k_{pol}$ values remained remarkably stable, ranging from 0.9962 to 1.0001. This study evaluated ion recombination and polarity effects in HDRE beams using plane parallel and cylindrical chambers.

**Conclusions:**

Based on data variations, we recommend elevated operating voltage (400 V) than typical voltage (300 V) to reduce ion recombination in HDRE beams. Furthermore, modified method can be alternative to measure the accuracy of charge readings for absolute dosimetry.

## 1 INTRODUCTION

The use of high dose rate electron beams (HDRE) in radiotherapy has been widely applied in Total Skin Electron Therapy (TSET) and Total Body Irradiation (TBI). Both techniques utilize HDRE beams on a linear accelerator (LINAC) with an extended source to skin distance (SSD) to achieve larger irradiation field and the ability to deliver high doses in a relatively short time than standard electron beam, thereby providing more optimal results.^1^, ^2^

Accurate radiation dose measurement is fundamental to ensuring the success of radiotherapy. In the context of HDRE beams, one of the primary challenges is the increased ionization density, which makes it more likely that ion recombination will occur within the ionization chamber compared to a standard electron beam. In detectors with a small sensitive volume, saturation is more likely to occur, potentially leading to inaccurate absorbed dose measurement.^3^, ^4^ In a related study, Kranzer et al., examined ion collection efficiency in Ultra High Dose Rate Electron (UHDR) using a custom designed plane parallel ionization chamber with electrode spacing varying from 0.5 to 2 mm, and reported that reducing electrode spacing can improve the ion collection efficiency.^5^

Extensive research has demonstrated the importance of ion recombination correction for obtaining reliable dosimetric data, especially when using standard dosimetry protocols such as IAEA TRS‐398 and AAPM TG‐51 methods. However, the commonly used two‐voltage method may not be sufficiently representative of HDRE conditions, as the higher ionization intensity requires a more thorough assessment of charge collection efficiency.^4^, ^6^, ^7^ The thesis research conducted by Pratiwi et al.^8^, investigated the calibration of electron beams in HDRE and standard modes using IAEA TRS‐398, AAPM TG‐51 and modified methods.^8^ The study showed that modified method demonstrated the lowest ion recombination correction factor $\left(k_{s}\right)$ discrepancy compared to IAEA TRS‐398 and AAPM TG‐51 dosimetry protocols. Furthermore, the results indicated that $k_{s}$ value in HDRE beams is 5.33% higher than standard electron beams.^8^ The modified methods introduced by Muir et al.^9^, involves adjusting the Effective Point of Measurement (EPOM) of the ionization chamber.^9^ At the same time, the beam quality conversion factor ($k_{Q}^{\prime }$) is derived by incorporating the power fitting parameters obtained from Monte Carlo simulations. In addition, these methods exclude the gradient correction factor applied in the AAPM TG‐51 protocol. However, the ion recombination analysis performed by Pratiwi et al. was limited to a single operating voltage (300 V), limiting the evaluation of systematic voltage variation on ion collection efficiency under HDRE conditions. Moreover, the combined evaluation of ion recombination and polarity effects in clinical HDRE beam has not been thoroughly investigated.

Referring to Pawiro et al,^10^ Mahfirotin et al,^11^ Yulinar et al.^12^, the use of HDRE beam is known to have several advantages. However, improper dose calibration procedures can cause significant side effects. Therefore, an accurate dosimetric approach is needed to ensure that the dose delivered conforms to the radiotherapy planning.^10^, ^11^, ^12^ Nevertheless, these studies mainly focused on applying modified calibration methods for standard clinical electron beams, with the main emphasis on evaluating electron beam output.

In order to address this gap, this study aims to systematically analyses the influence of ion recombination and polarity effects under voltage variations in HDRE using TRS‐398, AAPM TG‐51 and modified methods with three different ionization detectors (IBA CC13, FC65‐P and PPC40). In contrast to previous studies, this work evaluates ion recombination and polarity effects over a wider range of operating voltage (200–400 V) under clinical HDRE conditions. This approach allows a more comprehensive assessment of detector response and dosimetry accuracy, thereby providing practical recommendation for optimizing detector selection, chamber construction and operational voltage settings for the clinical implementation.

## 2 MATERIALS AND METHODS

The study was performed using Elekta Versa HD linear accelerator which generates nominal energy of 6 MeV and 10 MeV, while operating both in standard and HDRE modes with 100 MU. HDRE dose rate was in the range of 300–600 MU/s for both 6 HDRE MeV and 10 HDRE MeV electron beams. An 10×10cm applicator was applied for standard mode, while 40×40cm applicator was used for HDRE mode, as this is the only applicator size available for HDRE beam delivery.

### 2.1 Ionization chambers measurements

Three ionization chambers: plane parallel type (IBA PPC40 with SN 2389) and two cylindrical type (IBA CC13 with SN 20302 and FC65‐P with SN 6039) were employed for this work. The detailed specification of these chambers, as provided by manufacturer's technical datasheets are summarized in Table 1.^13^ Measurements were performed in 67.5×64.5×56 cm^3^ water phantom (Blue phantom, IBA), with the chambers positioned within the water as indicated in Table 2 for each method (TRS‐398, AAPM TG‐51 and modified method). During the study, electrometer Dose 1 (IBA) was used, with voltage varying from 100 to ± 400 V. To get a stable reading, all of the chambers were pre‐irradiated (initial warm‐up) prior to measurements at the beginning of each session.

**TABLE 1:** Characteristics of the ionization chamber as specified by the manufacturer.

Detector	Cavity volume ($cm^{3}$)	Cavity radius (mm)	Cavity length (mm)	Wall thickness ( 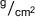 )	Length of inner electrode (mm)	Nominal voltage (V)
FC65‐P	0.65	3.1	23.1	0.057	20.5	300
CC13	0.13	3	5.8	0.07	3.3	300
Detector	Cavity volume ($cm^{3}$)	Entrance window diameter (mm)	Entrance window thickness (mm)	Collecting electrode diameter (mm)	Nominal voltage (V)	
PPC40	0.4	20	1	16	300	

**TABLE 2:** Placement depth ($Z_{\mathrm{placement}})$ of detectors according to AAPM TG‐51, TRS‐398 and modified method.

Detector	$Z_{placement}$(AAPM TG‐51)	$Z_{placement}$(TRS 398)	$Z_{placement}$(modified)
Cylindrical chamber	Central axis at $Z_{ref}$	Central axis at $Z_{ref}$ + $0.5r_{cavity}$	Central axis at $Z_{ref}$
Plane parallel chamber	EPOM at $Z_{ref}$—*window thickness*	EPOM at $Z_{ref}$—*window thickness*	EPOMat $Z_{ref}$ – 1.39 mm (*shifting muir)*

For all chambers, as recommended by AAPM TG‐51^14^ and TRS 398^15^ dosimetry protocols, the polarity correction $\left(k_{pol}\right)$ was determined by measuring the charge at both positive and negative polarizing voltages. Subsequently, all readings were recorded for each polarizing voltage, and then the chamber was given at least three minutes to ensure chamber equilibration before the next set of readings was taken.

In our study, ion recombination accounts for the loss of collection efficiency in ionization chambers. This correction is denoted $k_{s}$ in TRS‐398 and as $P_{ion}$ for AAPM TG‐51. The approach used in TRS‐398 is based on the linear relationship between the inverse of the chamber reading (1/M) and the operating voltage (1/V). Based on this relationship, value of this correction can be analyzed by measuring the chamber's reading at both the operating voltage (V_1_) and reduced voltage (V_2_),^16^ as expressed in Equation 1:

(1) $$k_{s}=a_{0}+a_{1}\left(\frac{M_{1}}{M_{2}}\right)+a_{2}{\left(\frac{M_{1}}{M_{2}}\right)}^{2}$$

where $M_{1}$ and $M_{2}$ are ion chamber readings at operating voltage (V_1_) and reduced voltage (V_2_) and $a_{0},\;a_{1},\;a_{2}$ are quadratic fit coefficients as function of voltage ratio (V_1_/V_2_). The determination of *k*_s_ values was carried out at five operating voltages of 200, 250, 300, 350, and 400 Volt with 100 Volt regarded as reduced voltage for each chamber used in study.

The AAPM TG‐51 protocol also uses the two‐voltage analysis (TVA) method to find $P_{ion}$ value, if ion recombination inside the ionization chamber follows Boag's theory, considering general recombination processes.^14^ As recommended by AAPM TG‐51 protocol, $P_{ion}$ value can be determined through the following Equation 2:

(2) $$\left(P_{ion}\left(V_{H}\right)\right)=\frac{1-(V_{H}/V_{L})}{M_{raw}^{H}/M_{raw}^{L}-V_{H}/V_{L}}\;$$

where $M_{raw}^{H}$ and $M_{raw}^{L}$ are chamber reading at normal voltage (V_H_) and lower voltage (V_L_). This method requires that the voltage ratio $V_{H}$ to $V_{L}$ must be no less than 2 to ensure valid determination.

For the modified method, the ion recombination value is determined using the same procedure as described in AAPM TG‐51 protocol, with the main difference being the detector placement. While AAPM TG‐51 positions the plane‐parallel chamber based on its physical thickness, the modified method applies the optimal shift that considers the effective point of measurement not always coincide with the geometric centre of chamber due to the limited size of the chamber cavity and the changing dose gradient. According to Muir's study 1.39 mm optimal shift was used for PPC40 in our study.^9^, ^12^

### 2.2 Absorbed dose to water measurement

Chamber readings at various applied voltages were collected for all detectors to calculate the absorbed dose using the dosimetry method described in the previous section. Although the primary objective is to investigate ion recombination and polarity effects in HDRE beams, the absolute dose to water calculation was used to validate the output results using electron beam absolute dosimetry formula, as detailed in AAPM TG‐51:

(3) $$D_{w,Q}\;\left(d_{ref}\right)=M_{Q}\;P_{gr}^{Q}K_{R50}^{\prime }K_{ecal}N_{D,w}^{Co}$$

where $P_{gr}^{Q}$ represent gradient correction factor for chamber, $K_{R50}^{\prime }$ is electron beam quality convertion, $K_{ecal}$ is beam quality conversion from photon to electron and $N_{D,w}^{Co}$ is the calibration factor for the absorbed dose in water. Furthermore, in modified method (Equation 4), the gradient correction factor was not explicitly calculated, instead, its implicitly incorporated into new beam quality factor $K_{Q}^{\prime }$ as presented in Equation (5) for plane parallel chamber and Equation (6) for cylindrical:^9^, ^10^, ^11^, ^17^

(4) $$D_{w,Q}\;\left(d_{ref}\right)=M_{Q}\;K_{Q}^{\prime }K_{Q,ecal}N_{D,w}^{Co},$$

(5) $${k^{\prime }}_{Q}\;\left(pp\right)=a+b\times e^{-\frac{R_{50}}{c}},$$

(6) $${k^{\prime }}_{Q}\;\left(cyl\right)=a+b\times R_{50}^{-c},$$

where a,b and c are exponential fitting parameter for $k_{Q}^{\prime }$.^9^

## 3 RESULTS

### 3.1 Polarity effect

The $k_{pol}$ values for all understudy chambers at different electron energies shows minimum polarity effect (0.9962–1.0001), with no significant impact from the increase of bias voltage. PPC40 showed the best performance with smallest deviation (0.0300–0.2199 %) to the reference value, followed by FC65‐P (0.0100–0.2301%) and CC13 (0.0901–0.39%) that shown in Tables 3 and 4. The reference value in this study corresponds to measurements obtained using the standard electron beam with 10 × 10 cm applicator. These results indicate that the polarity effect becomes more significant as the electrode spacing decreases due to stronger electric field gradients within the chamber.

**TABLE 3:** Comparison of $k_{pol}$ values by different protocol dosimetry for PPC40, FC65‐P and CC13 at various operating voltage at 6 MeV in standard and HDRE.

Operating voltage (V)	Detector	6 MeV (10 × 10 cm)	6 MeV HDRE (40 × 40 cm)					
		Polarity correction values ($K_{pol}$)						
		TRS 398	TRS 398	Deviation (%)	TG 51	Deviation (%)	Modified	Deviation (%)
200	PPC40	–	1.0000	−0.0300	0.9996	−0.0700	0.9985	−0.1799
250	PPC40	–	0.9999	−0.0400	0.9999	−0.0400	0.9982	−0.2099
300	PPC40	1.0003	1.0000	−0.0300	0.9997	−0.0600	1.0000	−0.0300
350	PPC40	–	0.9997	−0.0600	0.9997	−0.0600	1.0000	−0.0300
400	PPC40	–	0.9999	−0.0400	0.9999	−0.0400	1.0000	−0.0300
200	CC13	–	0.9962	−0.3900	0.9963	−0.3800	0.9963	−0.3800
250	CC13	–	0.9963	−0.3800	0.9963	−0.3800	0.9963	−0.3800
300	CC13	1.0001	0.9977	−0.2400	0.9967	−0.3400	0.9967	−0.3400
350	CC13	–	0.9964	−0.3700	0.9967	−0.3400	0.9967	−0.3400
400	CC13	–	0.9963	−0.3800	0.9964	−0.3700	0.9964	−0.3700
200	FC65‐P	–	0.9992	0.0902	0.9978	−0.0501	0.9978	−0.0501
250	FC65‐P	–	0.9991	0.0801	0.9991	0.0801	0.9991	0.0801
300	FC65‐P	0.9983	0.9988	0.0501	0.9978	−0.0501	0.9978	−0.0501
350	FC65‐P	–	0.9988	0.0501	0.9978	−0.0501	0.9978	−0.0501
400	FC65‐P	–	0.9987	0.0401	0.9982	−0.0100	0.9982	−0.0100

**TABLE 4:** Comparison of $k_{pol}$ values by different protocol dosimetry for PPC40, FC65‐P and CC13 at various operating voltage at 10 MeV in standard and HDRE.

Operating voltage (V)	Detector	10 MeV (10 × 10 cm)	10 MeV HDRE (40 × 40 cm)					
		Polarity correction values ($K_{pol}$)						
		TRS 398	TRS 398	Deviation (%)	TG 51	Deviation (%)	Modified	Deviation (%)
200	PPC40	–	1.0001	−0.0300	1.0001	−0.0300	0.9982	−0.2199
250	PPC40	–	0.9999	−0.0500	0.9997	−0.0700	0.9989	−0.1499
300	PPC40	1.0004	0.9999	−0.0500	1.0000	−0.0400	1.0001	−0.0300
350	PPC40	–	0.9997	−0.0700	1.0000	−0.0400	0.9999	−0.0500
400	PPC40	–	1.0000	−0.0400	0.9999	−0.0500	0.9999	−0.0500
200	CC13	–	0.9970	−0.1702	0.9976	−0.1101	0.9976	−0.1101
250	CC13	–	0.9969	−0.1802	0.9976	−0.1101	0.9976	−0.1101
300	CC13	0.9987	0.9973	−0.1402	0.9979	−0.0801	0.9979	−0.0801
350	CC13	–	0.9967	−0.2003	0.9971	−0.1602	0.9971	−0.1602
400	CC13	–	0.9966	−0.2103	0.9975	−0.1202	0.9975	−0.1202
200	FC65‐P	–	0.9986	−0.0800	0.9988	−0.0600	0.9988	−0.0600
250	FC65‐P	–	0.9991	−0.0300	0.9987	−0.0700	0.9987	−0.0700
300	FC65‐P	0.9994	1.0002	0.0800	0.9987	−0.0700	0.9987	−0.0700
350	FC65‐P	–	0.9995	0.0100	0.9989	−0.0500	0.9989	−0.0500
400	FC65‐P	–	0.9990	−0.0400	0.9971	−0.2301	0.9971	−0.2301

### 3.2 Ion recombination

**FIGURE 1:**
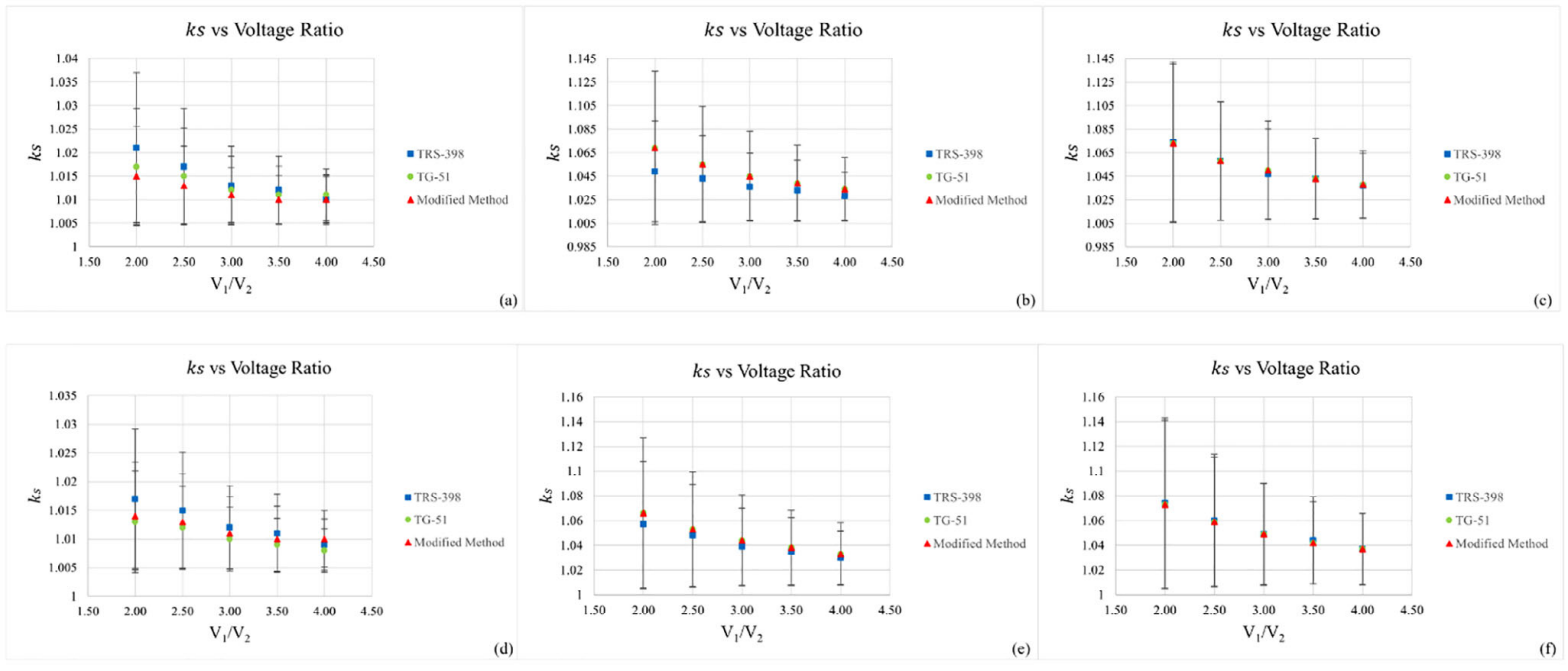
Relationship between applied voltage (V1/V2) and ion collection efficiency $\left(k_{s}\right)$ for PPC40, CC13 and FC65‐P ionization chambers under HDRE conditions at 6 MeV (a–c) and 10 MeV (d–f).

The results of ion recombination $\left(k_{s}\right)$ value evaluation through different methods for various operating voltage ranging from 200 to 400 V are summarized in Tables 5 and 6. The evaluation showed decreasing trend of $k_{s}$ value as the voltage increased, as illustrated in Figure 1 indicating that stronger electric fields contribute to better charge collection efficiency. In particular, the plane parallel PPC40 chamber showed significant stability across all three methods, with the maximum deviation of 1.5622 at 200 V. In contrast, FC65‐P and CC13 recorded $k_{s}$ value of 1.0740 and 1.0692 above recommended by the protocol $\left(k_{s}<1.05\right)$ at 200 V, indicating significant recombination issues. This finding are in line with the previous work performed by Baghani et al., and Kranzer et al., due to low mobility of ions at low voltages, combined with the longer electrode spacing in the cylindrical design, which collectively increase the probability of recombination in the case of high dose per pulse intraoperative and ultra‐high dose per pulse in electron beam.^5^, ^18^

**TABLE 5:** Comparison of $k_{s}$ values by different protocol dosimetry for PPC40, FC65‐P and CC13 at various operating voltage at 6 MeV in standard and HDRE.

Operating voltage ratio (V_1_/V_2_)	Detector	6 MeV (10 × 10 cm)	6 MeV HDRE (40 × 40 cm)					
		Ion recombination correction values ($k_{s}$)						
		TRS 398	TRS 398	Deviation (%)	TG 51	Deviation (%)	Modified	Deviation (%)
200/100	PPC40	–	1.0207	1.5622	1.0172	1.2139	1.0154	1.0348
250/100	PPC40	–	1.0172	1.2139	1.0150	0.9950	1.0133	0.8259
300/100	PPC40	1.0050	1.0133	0.8259	1.0122	0.7164	1.0108	0.5771
350/100	PPC40	–	1.0123	0.7164	1.0111	0.6070	1.0101	0.5075
400/100	PPC40	–	1.0102	0.5174	1.0105	0.5473	1.0099	0.4876
200/100	CC13	–	1.0491	4.0980	1.0692	6.0925	1.0692	6.0925
250/100	CC13	–	1.0433	3.5225	1.0551	4.6934	1.0551	4.6934
300/100	CC13	1.0078	1.0355	2.7486	1.0446	3.6515	1.0446	3.6515
350/100	CC13	–	1.0330	2.5005	1.0393	3.1256	1.0393	3.1256
400/100	CC13	–	1.0278	1.9845	1.0341	2.6096	1.0341	2.6096
200/100	FC65‐P	–	1.0735	6.2977	1.0733	6.2778	1.0733	6.2778
250/100	FC65‐P	–	1.0581	4.7727	1.0579	4.7529	1.0579	4.7529
300/100	FC65‐P	1.0099	1.0467	3.6439	1.0503	4.0004	1.0503	4.0004
350/100	FC65‐P	–	1.0426	3.2379	1.0432	3.2974	1.0432	3.2974
400/100	FC65‐P	–	1.0365	2.6339	1.0377	2.7527	1.0377	2.7527

**TABLE 6:** Comparison of $k_{s}$ values by different protocol dosimetry for PPC40, FC65‐P and CC13 at various operating voltage at 10 MeV in standard and HDRE.

Operating voltage Ratio (V_1_/V_2_)	Detector	10 MeV (10 × 10 cm)	10 MeV HDRE (40 × 40 cm)					
		Ion recombination correction values ($k_{s}$)						
		TRS 398	TRS 398	Deviation (%)	TG 51	Deviation (%)	Modified	Deviation (%)
200/100	PPC40	–	1.0166	1.1945	1.0134	0.8760	1.0139	0.9257
250/100	PPC40	–	1.0146	0.9954	1.0117	0.7067	1.0129	0.8262
300/100	PPC40	1.0046	1.0118	0.7167	1.0102	0.5574	1.0109	0.6271
350/100	PPC40	–	1.0114	0.6769	1.0092	0.4579	1.0103	0.5674
400/100	PPC40	–	1.0091	0.4479	1.0084	0.3783	1.0095	0.4878
200/100	CC13	–	1.0571	4.8294	1.0661	5.7219	1.0661	5.7219
250/100	CC13	–	1.0481	3.9369	1.0529	4.4129	1.0529	4.4129
300/100	CC13	1.0084	1.0388	3.0147	1.0440	3.5303	1.0440	3.5303
350/100	CC13	–	1.0353	2.6676	1.0377	2.9056	1.0377	2.9056
400/100	CC13	–	1.0297	2.1123	1.0329	2.4296	1.0329	2.4296
200/100	FC65‐P	–	1.0740	6.3998	1.0734	6.3404	1.0734	6.3404
250/100	FC65‐P	–	1.0602	5.0327	1.0590	4.9138	1.0590	4.9138
300/100	FC65‐P	1.0094	1.0488	3.9033	1.0492	3.9429	1.0492	3.9429
350/100	FC65‐P	–	1.0435	3.3782	1.0417	3.1999	1.0417	3.1999
400/100	FC65‐P	–	1.0373	2.7640	1.0374	2.7739	1.0374	2.7739

**FIGURE 2:**
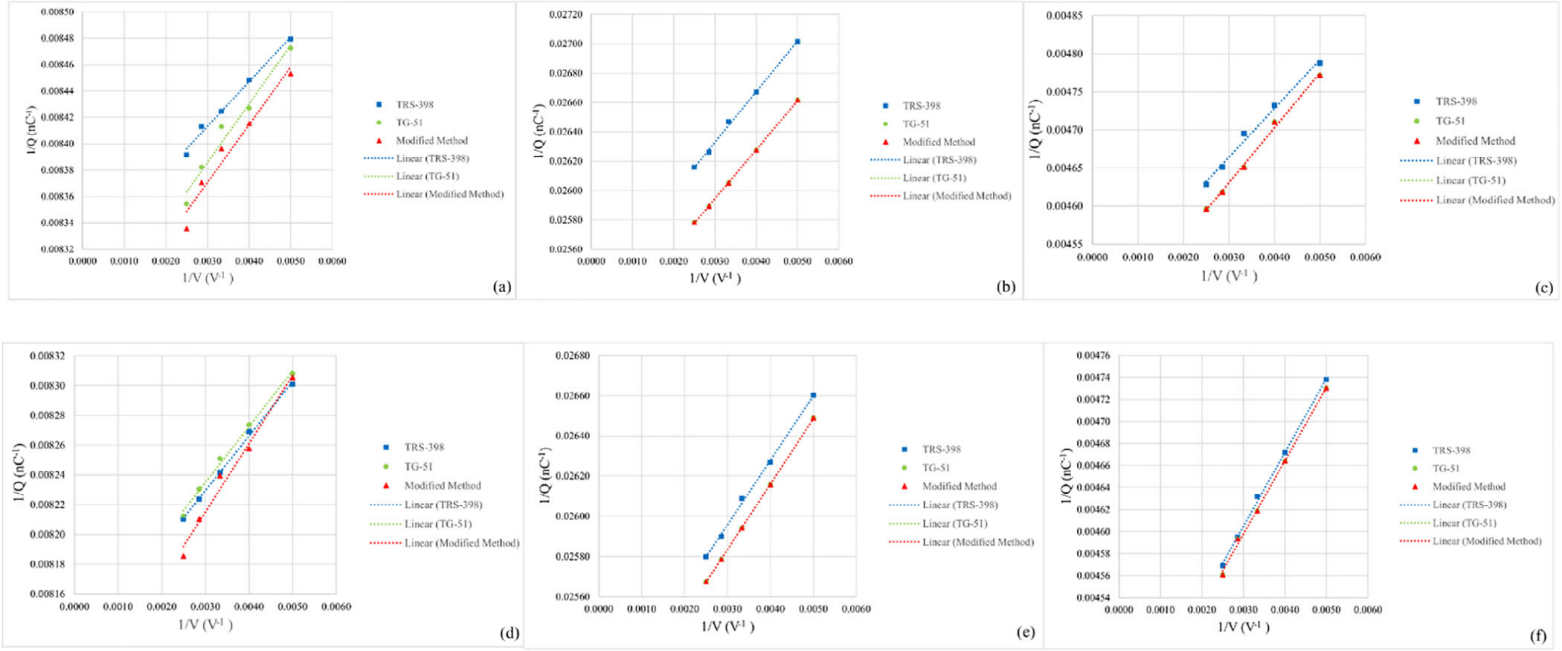
Jaffé plot showing the relationship between 1/Q and 1/V for PPC40, CC13 and FC65‐P ionization chambers under HDRE condition at 6 MeV (a–c) and 10 MeV (d–f). The solid symbols (squares, circles and triangles) represent the measured chamber reading, while the dashed lines indicate the corresponding linear interpolations.

The validity of the ion recombination correction measurements for the HDRE beam was validated using Jaffé plots generated for all three calibration methods and three ionization chambers (PPC40, CC13 and FC65‐P) as seen in Figure 2. All plots demonstrated a clear linear relationship, with coefficients of determination (*R* ^2^) ranging from 0.9546 to 1.000 as presented in Table 7. This strong linearity confirms that ion recombination predictable behavior under HDRE conditions, thereby supporting the reliability of the applied correction methods.

**FIGURE 3:**
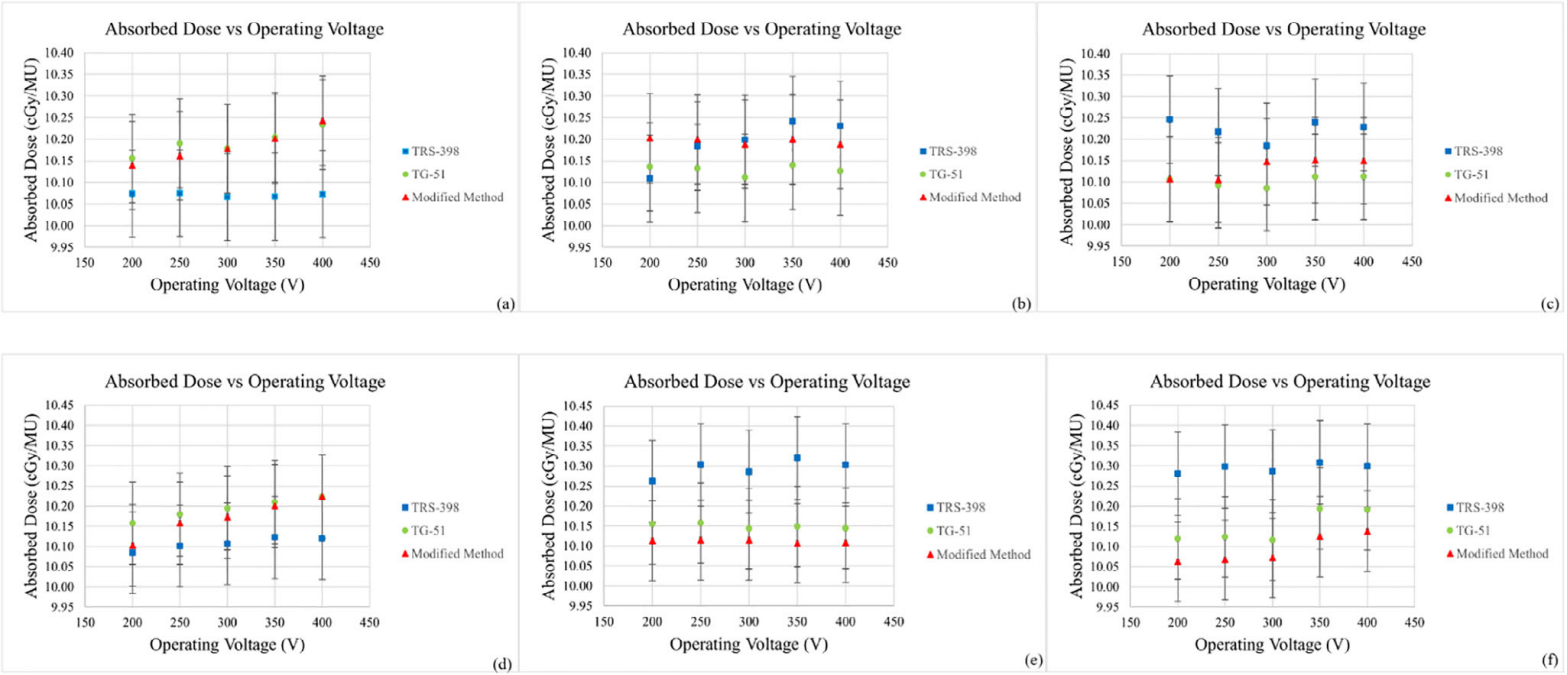
Absolute absorbed dose (cGy/MU) obtained using three dosimetry methods with PPC40, CC13 and FC65‐P ionization chambers under HDRE condition at 6 MeV (a–c) and 10 MeV (d–f).

**TABLE 7:** Coefficient of determination (*R* ^2^) values obtained from the linear fitting of Jaffe plots for PPC40, FC65‐P and CC13 ionization chamber under HDRE conditions at 6 MeV and 10 MeV electron beams.

Energy	Detector	Jaffe plot (R^2^)		
		TRS‐398	TG‐51	Modified
6 MeV HDRE	PPC40	0.9901	0.9677	0.9546
	FC65‐P	0.9907	0.9958	0.9958
	CC13	0.9978	0.9997	0.9997
10 MeV HDRE	PPC40	0.9976	0.9943	0.9833
	FC65‐P	0.9984	0.9975	0.9975
	CC13	0.9980	1.0000	1.0000

### 3.3 Absorbed dose to water

The measurement results showed variations in absorbed dose values ​​obtained using each calibration method, for 6 MeV and 10 MeV HDRE, depending on the type of detector used and the applied operating voltage as seen in Figure 3. In summary, the modified method provides absorbed dose values ​​close to the AAPM TG‐51 method, while the most significant differences are seen between the TRS‐398 and TG‐51 methods, especially at low energies (6 MeV HDRE). These differences can be attributed to the theoretical approach and parameter corrections used by each method. The TRS‐398 uses a calibration factor in units of absorbed dose in air with a $k_{Q}$ beam quality correction factor. At the same time, the TG‐51 applies measurements at two different depths to calculate the electron dose gradient. Meanwhile, the modified method uses a ${k^{\prime }}_{Q}$ beam quality conversion factor from the Monte Carlo calculation results that implicitly calculated electron dose gradient.

For the plane‐parallel detector (PPC40), the modified method and TG‐51 results in slightly higher absorbed dose values ​​compared to TRS‐398, with deviation ratios of approximately 1.0–1.016 at 6 MeV and 1.0–1.014 at 10 MeV. This difference may arise as in clinic, dose readings are typically adjusted according to the correction factors specified in the TRS‐398 protocol, which may lead to a slightly lower absorbed dose estimate.

Overall, the modified method provides comparable results to international protocols (IAEA TRS‐398 and AAPM TG‐51), while offering simpler procedure by removing the dual depth requirements of TG‐51, thereby reducing positioning uncertainties and improving clinical efficiency.

## 4 DISCUSSION

This study investigates the impact of polarity and ion recombination effect for three commercial ionization detectors used in HDRE beam. As described in the results section, $k_{pol}$ for PPC40 has the value of 1, which indicates the smallest polarity effect compared to FC65‐P and CC13. The PPC40's greater ability to reduce polarity effects can be due to its geometric design, which has a guard ring.^5^ The guard ring in PPC40 effectively reduces edge effects. At the same time, its geometric design provides a large and uniform collecting area, thereby minimizing electric field gradient within the sensitive volume and reducing polarity dependence. Shorter electrode spacing produces a higher electric field intensity and gradient, which can influence charge collection and recombination processes. However, the polarity dependence observed in the cylindrical detectors like FC65‐P and CC13 is more likely from interactions outside the sensitive volume. This effect becomes more noticeable in chambers with smaller sensitive volumes.^19^

During this study, no consistent relationship was found regarding applied voltage and polarity effect. These results are in line with Looe et al., study, which confirmed that voltage adjustments do not have a significant impact on the polarity effect. Instead, detectors geometries mainly the size of its sensitive volume are considered to have more influence on the dispersion and uniformity of the electric field.^3^

For ion recombination effect, the increase in operational voltage is directly correlated with the strengthening of the electric field, which significantly accelerates the movement of ions towards the collector electrode. Consequently, the charge collection time in the sensitive volume of the detector becomes shorter, thereby reducing the possibility of recombination between the formed ion pairs.^7^, ^16^, ^18^ Our evaluation, which are consistent with previous data in Tables 6 and 7, show that operating the PPC40, CC13, and FC65‐P detectors at a voltage of 400 V despite exceeding the manufacturer's recommendation of 300 V effectively lowers the value of the recombination correction factor. This behaviour is further supported by the high degree of linearity observed in the Jaffé plots, which confirms that the relationship between 1/Q and 1/V is well described by the Jaffé model and validates the applicability of the two voltage method under HDRE condition. Similar findings have been reported in studies of Ultra High Dose Rate (UHDR) and Flash electron beams, where strong electric field are required to maintain accurate charge collection in ionization chambers exposed to high instantaneous dose rates. These comparisons suggest that detector behaviour under HDRE conditions shares similar physical mechanisms with other high dose‐rate electron beam modalities.^4^, ^5^ These findings can be used for determining the optimal voltage and selecting the most suitable detector type and construction for clinical application of HDRE beams in the ion chamber tested in this study. In addition, variations in reading values at different operating voltages (200–400 V) indicate that increasing the voltage enhances charge collection efficiency; however, between 350 and 400 V, the changes become relatively small, suggesting that the detector is approaching saturation. Nevertheless, further investigation is required at an operating voltage of 500 V the maximum voltage limit for the PPC40, FC65‐P, and CC13 detectors to determine whether charge collection becomes more efficient or if the detectors have already reached saturation.

On the other hand, as previously discussed regarding the detector's geometric characteristics and electrode configuration of the detector, the charge collection efficiency is also greatly influenced by the physical properties of the detector, especially the inter‐electrode spacing and size of sensitive volume. Detectors with larger sensitive volumes and electrode spacing typically have higher recombination rates. This is due to the longer ion travel time towards the collector electrode, the lower charge collection efficiency, and the higher ion recombination value.^5^, ^20^, ^21^

Furthermore, the application of applying modified method with a 1.39 mm EPOM shift in detector PPC40 demonstrated a smaller deviation compared to the IAEA TRS 398 and comparable to the AAPM TG‐51 protocol in the measurement of ion recombination correction factor. In addition, the use of ${k^{\prime }}_{Q}$ factor derived from Monte Carlo calculations in the modified method yielded absorbed dose values comparable to those obtained using both reference protocols. These findings indicate that modified method provides consistent and accurate calibration results.

Therefore, this approach is a potential alternative for HDRE beam dose calibration, particularly because its simplified formulation eliminated the need for measurements at two different depths, as required in AAPM TG‐51 protocol, without compromising accuracy. Moreover, this simplification improves time efficiency and reduces potential uncertainties arising from positional variation, making it a practical method for clinical implementation.

## 5 CONCLUSION

The performance of plane parallel (PPC40) and cylindrical (FC65‐P and CC13) chambers was evaluated for ion recombination and polarity effect through different recommended methods at various operating voltages. Our study indicates that ion recombination is strongly dependent on the applied voltage, while the polarity effect shows no significant dependence.

The ion recombination effect is consistently higher in the cylindrical ionization chamber compared to the parallel plate ionization chamber, which may be related to the difference in detector geometric construction. In parallel plate detectors, the electrodes are arranged parallel in parallel configuration, resulting in an electric field is nearly uniform across the sensitive volume. Furthermore, the smaller electrode spacing and active volume reduce the time required for ions and electrons to reach the electrodes, allowing the electric field to act more uniformly and efficiently in charge collection. Overall, PPC40 demonstrated the most stability and measurement accuracy across various correction factors, as well as in HDRE beam absolute dosimetry, compared to FC65‐P and CC13.

## AUTHOR CONTRIBUTIONS

Ratna Sari Dewi was responsible in data collection, data analysis, and manuscript writing. Mardon Limena contributed in data collection. Dwi Seno Kuncoro Sihono contributed in data analysis. Poonam Yadav contributed in data analysis and manuscript writing. Supriyanto A. Pawiro was responsible in research design, data collection, data analysis, and manuscript writing.

## CONFLICT OF INTEREST STATEMENT

The authors declare no conflicts of interest.

## ETHICAL APPROVAL

No human subjects were involved in this study.

## Data Availability

The data that support the findings of this study are available from the corresponding author upon reasonable request.
